# Editorial: Psychiatric comorbidities of neurogenetic and neurodegenerative diseases

**DOI:** 10.3389/fneur.2026.1930461

**Published:** 2026-07-28

**Authors:** Rui Ban, Li Wang, Xiaohui Duan, Kai Dong, Anthony L. Vaccarino

**Affiliations:** 1 Department of Neurology, The First Hospital of Tsinghua University, School of Clinical Medicine, Tsinghua Medicine, Tsinghua University, Beijing, China; 2 Department of Neurology, China-Japan Friendship Hospital, Beijing, China; 3 Department of Neurology, Beijing Anding Hospital, Capital Medical University, Beijing, China; 4 Indoc Research, Toronto, ON, Canada

**Keywords:** ADHD, CADASIL, chorea-acanthocytosis, cognitive impairment, pantothenate kinase-associated neurodegeneration, Parkinson's disease, primary coenzyme Q10 deficiency, Wilson's disease

Neurogenetic and neurodegenerative diseases have long been framed as disorders of motor control, cognition, or sensorimotor integration. With the global increase in life expectancy, the prevalence of neurodegenerative diseases such as Alzheimer's disease (AD), Parkinson's disease (PD), Motor neuron disease (MND), and Frontotemporal lobe degeneration (FLD) is rising, placing significant burdens on patients, families, and healthcare systems. While some progress has been made in understanding the pathogenesis of these diseases, their exact etiologies involving genetic, environmental, and lifestyle factors have not yet been fully elucidated, presenting ongoing challenges in treatment and management. Yet, for patients and families, some of the most disabling features are often psychiatric: depression, anxiety, severe sleep disturbances, behavioral shifts, psychosis, compulsive behaviors, and neurodevelopmental comorbidities such as ADHD and autism spectrum traits. These manifestations often appear early, persist stubbornly, and significantly impact quality of life. However, they are frequently under-recognized, undertreated, and insufficiently integrated into both research and clinical pathways.

This Research Topic bridges neurological and psychiatric disorders through a shared mechanistic and clinical agenda, addressing conditions that often co-occur and complicate patient management. This topic seeks to improve diagnostics, therapeutics, and ultimately patient prognosis through an interdisciplinary approach. The contributions compiled herein—spanning rare neurogenetic syndromes, common neurodegenerative diseases, and neurodevelopmental disorders—underscore the frequency with which “neurological” and “psychiatric” phenotypes co-occur and share pathophysiological roots.

The Research Topic “*Psychiatric comorbidities of neurogenetic and neurodegenerative diseases*” features 10 studies (two reviews, two case reports, one Brief Research Report, and five original research articles) spanning rare genetic disorders, Parkinson's disease (PD), neurodevelopmental impairments, and cognitive dysfunction. These works bridge basic research, clinical observation, and translational insights, highlighting the intricate interplay between nutrition, genetics, neural circuits, and systemic factors in neurological health and disease.

## Rare genetic neurological disorders: decoding pathogenesis and phenotypic diversity

Rare genetic neurological diseases, often underdiagnosed due to phenotypic heterogeneity, offer profound insights into basic neural mechanisms. Five studies in this Research Topic focus on distinct rare genetic disorders, uncovering novel mutations and pathophysiological pathways, and the nuances of therapeutic windows.

Wang et al. (“*CADASIL or NOTCH3 mutation spectrum diseases? Interpretation of NOTCH3 mutations and clinical heterogeneity in CADASIL*”) provide a thought-provoking review that reframes CADASIL as part of a broader NOTCH3 mutation spectrum rather than a single disease entity. Their work highlights the remarkable clinical heterogeneity associated with NOTCH3 variants, emphasizing that psychiatric manifestations—including mood disorders, apathy, and cognitive impairment—may precede overt cerebrovascular events in some patients. By expanding the recognized phenotypic spectrum beyond recurrent ischemic stroke and white matter lesions, the authors underscore the importance of considering inherited cerebral small vessel disease in patients presenting with otherwise unexplained neuropsychiatric symptoms. This broader perspective has important implications for earlier recognition, genetic diagnosis, and individualized management of NOTCH3-related disorders. Psychiatric symptoms may therefore represent an early clinical clue to underlying cerebral small vessel pathology rather than merely secondary consequences of vascular injury.

This theme of genetic and phenotypic heterogeneity is further reinforced by studies on rare movement disorders. Chorea-acanthocytosis (ChAc) is the most common subtype of neuroacanthocytosis (NA) caused by mutations in VPS13A (vacuole protein sorting-associated protein 13A). Lin et al. (“*Novel loss-of-function mutations in VPS13A cause chorea-acanthocytosis in two families*”) identified novel loss-of-function mutations in VPS13A causing chorea-acanthocytosis (ChAc), expanding the genetic landscape and emphasizing the systemic nature of chorein deficiency—which can manifest in unexpected features like alopecia. They reported families with VPS13A mutations who had not just progressive movement disorders and dementia, but also prominent neuropsychiatric features alongside distinctive red blood cell shapes. This shared membrane pathology—affecting neurons and erythrocytes alike—suggests that the psychiatric and cognitive problems are not downstream consequences of neurodegeneration. They are part of the same cellular instability. Meanwhile, Liang et al. (“*Case report: clinical manifestations and imaging features associated with PANK2 c.940C*>*T variant in PKAN with symmetric basal ganglia calcification*”) reported an atypical case of pantothenate kinase–associated neurodegeneration (PKAN) and provides the first detailed genotype–phenotype (dystonia, acanthocytosis, and cognitive decline) correlation for this locus.

Wu et al. (“*IDEDNIK syndrome: a newly recognized rare genetic disorder caused by AP1S1 and AP1B1 mutations*”) provide a critical update on IDEDNIK syndrome, a rare autosomal recessive neurocutaneous disorder caused by AP1S1 or AP1B1 mutations. Their work highlights how a defect in intracellular vesicle trafficking can disrupt copper homeostasis, creating a phenotype that overlaps with both Menkes and Wilson's diseases, yet demands a distinct multidisciplinary management approach. The review details clinical diagnosis, genetic testing, and emerging therapies (e.g., oral zinc acetate). This work underscores the importance of recognizing rare neurocutaneous disorders and expanding genetic screening for atypical presentations.

Wang et al. (“*Case report: myoclonic and tremulous movements associated with COQ8A-related coenzyme Q10 deficiency type 4*”) present a compelling case report of COQ8A-related CoQ10 deficiency type 4, a mitochondrial disorder typically known for cerebellar ataxia. They describe a young patient whose myoclonic tremor, slurred speech, and gait disturbance were accompanied by emotional storms that waxed and waned with neurological crises. The psychiatric instability wasn't incidental—it tracked with the underlying bioenergetic failure. Notably, targeted CoQ10 supplementation reversed both motor and affective symptoms, supporting the hypothesis that early intervention at the metabolic level can lead to the improvement of psychiatric manifestations. This aligns with broader recognition that mitochondrial dysfunction does not respect categorical boundaries between neurology and psychiatry (1), highlighting that treatable mitochondrial disorders can masquerade as common movement disorders. The rapid genetic diagnosis and subsequent clinical stabilization with CoQ10 supplementation underscore the therapeutic imperative of considering these etiologies early.

Beyond rare genetic disorders, similar mechanistic principles extend to neurodevelopmental conditions. This Research Topic further highlights the value of advanced imaging and mechanistic modeling in understanding common neurodevelopmental disorders. Li et al. (“*Impaired glymphatic system function and its association with speech and language delay in children with ADHD: a prospective study*”) provide a groundbreaking prospective study linking the glymphatic system to Attention-Deficit/Hyperactivity Disorder (ADHD). Diffusion tensor image analysis along the perivascular space (DTI-ALPS) was used to calculate the ALPS index, which evaluates impaired glymphatic function in children with ADHD. They found impaired brain waste clearance in this pediatric cohort, with a specific link to speech and language delay. This offers a novel perspective on neurodevelopmental psychiatric conditions, framing them not merely as issues of neural circuitry or neurochemistry, but as deficits in glymphatic clearance mechanisms during critical developmental windows. The glymphatic system—essentially the brain's primary mechanism for clearing metabolic waste products—has been implicated in neurodegeneration and dementia (2–4). This suggests that impaired waste clearance—a mechanism classically associated with neurodegeneration—may also play a role in neurodevelopmental delay, opening new avenues for understanding comorbidities in ADHD.

## Parkinson's disease and dystonia: nutrition, sleep, cognitive impairment, and gait—Interconnected non-motor and motor domains

This concept of network-level dysfunction is further exemplified in neurodegenerative disorders such as Parkinson's disease. Across these studies, a coherent picture emerges of brain networks under stress—systems regulating emotion, sleep, cognition, and behavior failing alongside motor and cognitive circuits. The mechanisms vary: including mitochondrial collapse, membrane instability, vascular fragility, protein misfolding, defective waste clearance. But the outcome is consistent: disruption ripples through interconnected circuits, and psychiatric features become not just complications but markers of disease activity, progression, and even treatment response.

In Parkinson's disease, the interplay gets even more complex. Zhang et al. (“*Stage-specific efficacy of pharmacological interventions on gait impairments in Parkinson's disease*”) showed that objective gait measures worsen across disease stages and respond, at least partially, to dopaminergic therapy—especially in advanced disease. Meanwhile, Yang et al. (“The *relationship between nutritional status and sleep quality in Parkinson's disease: a single tertiary center study*”) documented just how common malnutrition and sleep disturbance are in PD, and how tightly these correlate with motor severity and biochemical markers like total protein. Sleep deprivation and poor nutrition do not merely induce fatigue and physical weakness; they exacerbate mood disturbances, diminish motivation, and impair cognitive clarity. Thus, the neuropsychiatric burden in PD is not solely attributable to dopamine loss; rather, it is shaped by systemic factors that feed back into brain function (5).

Li et al. (“*Cognitive impairment in Chinese patients with isolated generalized dystonia: a case-control study*”) presented a case-control study investigating cognitive function in generalized dystonia (GD) patients, finding poorer performance in MoCA, executive function/attention, spatial ability, and some memory tests vs. healthy controls. Some cognitive impairments correlated with motor severity and psychiatric issues, but medication and etiologies of dystonia did not influence cognition. The study suggests cognitive impairment may be a GD manifestation partly linked to motor and psychiatric factors.

Even hypertension, not traditionally thought of as a “brain disease,” gets reconsidered here. Wang et al. (“*Correlation study between serum uric acid level and cognitive impairment in middle-aged and elderly patients with hypertension: based on an explainable predictive model*”) used machine learning to explore serum uric acid levels in hypertensive patients and found a U-shaped relationship with mild cognitive impairment. Both deficient and elevated levels of this antioxidant appear detrimental to cognition, situating cognitive decline within a metabolic-vascular framework where oxidative stress and inflammation modulate neural vulnerability. Collectively, these findings reinforce the notion that psychiatric and cognitive manifestations consistently track disease burden across both rare and common neurological disorders.

So what does all this add up to? Psychiatric comorbidities aren't peripheral—they're central to understanding how these diseases work. Whether the insult is energetic, structural, vascular, or metabolic, the resulting network dysfunction doesn't confine itself to motor or “purely cognitive” domains. Emotion, behavior, sleep, and development are vulnerable too, and their dysfunction can be just as revealing—and just as treatable. We need to actively look for and track psychiatric symptoms, not treat them as soft endpoints. Advances in imaging, genomics, and biomarkers now let us connect these symptoms to specific biological mechanisms, enabling more precise diagnosis and targeted intervention. And many of these conditions—CoQ10 deficiency, vascular-metabolic cognitive impairment—are modifiable, especially if caught early.

The future of neurology isn't about policing the boundary between “neurological” and “psychiatric.” It's about recognizing that these are artificial distinctions that fragment our understanding of brain disease. The articles in this Research Topic point toward a more unified framework—one rooted in network biology, attentive to the full spectrum of human experience, and committed to care that's not just more effective, but more humane.

## References

[1] Pinto Payares DV Spooner L Vosters J Dominguez S Patrick L Harris A . A systematic review on the role of mitochondrial dysfunction/disorders in neurodevelopmental disorders and psychiatric/behavioral disorders. Front Psychiatry. (2024) 15:1389093. doi: 10.3389/fpsyt.2024.1389093 39006821 PMC11239503

[2] Hladky SB Barrand MA. The glymphatic hypothesis: the theory and the evidence. Fluids Barriers CNS. (2022) 19:9. doi: 10.1186/s12987-021-00282-z 35115036 PMC8815211

[3] Barlattani T Grandinetti P Cintio AD Montemagno A Testa R D'Amelio C . Glymphatic system and psychiatric disorders: a rapid comprehensive scoping review. Curr Neuropharmacol. (2024) 22, 2016–33. doi: 10.2174/1570159X22666240130091235 39234773 PMC11333792

[4] Corbali O Levey AI. Glymphatic system in neurological disorders and implications for brain health. Front Neurol. (2025) 16:1543725. doi: 10.3389/fneur.2025.1543725 39974364 PMC11835678

[5] Howarth NE Miller MA. Sleep, sleep disorders, and mental health: a narrative review. Heart Mind. (2024) 8, 146–58. doi: 10.4103/hm.HM-D-24-00030

